# Protective Evaluation of Compounds Extracted from Root of *Rhodiola rosea* L. against Methylglyoxal-Induced Toxicity in a Neuronal Cell Line

**DOI:** 10.3390/molecules25122801

**Published:** 2020-06-17

**Authors:** Cheng-Hao Wang, Safwan Safwan, Min-Chi Cheng, Te-Yu Liao, Lin-Chen Cheng, Ting-An Chen, Yueh-Hsiung Kuo, Yung-Feng Lin, Ching-Kuo Lee

**Affiliations:** 1 Graduate Institute of Pharmacognosy, Taipei Medical University, Taipei 11031, Taiwan; tnssh31833@hotmail.com; 2 Ph.D. Program in Clinical Drug Development of Herbal Medicine, Taipei Medical University, Taipei 11031, Taiwan; safwan_afan@yahoo.com; 3 Faculty of Health Science, University of Muhammadiyah Mataram, Mataram 83127, Indonesia; 4 School of Pharmacy, Taipei Medical University, Taipei 11031, Taiwan; d301100008@tmu.edu.tw (M.-C.C.); b313106079@tmu.edu.tw (T.-Y.L.); b313106083@tmu.edu.tw (L.-C.C.); b313106068@tmu.edu.tw (T.-A.C.); 5 Department of Chinese Pharmaceutical Sciences and Chinese Medicine Resources, College of Pharmacy, China Medical University, Taichung 40402, Taiwan; kuoyh@mail.cmu.edu.tw; 6 School of Medical Laboratory Science and Biotechnology, College of Medical Science and Technology, Taipei Medical University, Taipei 11031, Taiwan; 7 Ph.D. Program in Medical Biotechnology, College of Medical Science and Technology, Taipei Medical University, Taipei 11031, Taiwan

**Keywords:** *Rhodiola rosea*, neuroprotective activity, acetylcholinesterase, methylglyoxal, neuro-2A

## Abstract

*Rhodiola rosea* L. (*R. rosea*) is one of the most beneficial medicinal plants and it is studied as an adaptogen. This study aims to evaluate the neuroprotective activity of compounds extracted from the root of *R. rosea* against methylglyoxal (MG)-induced apoptosis in neuro-2A (N2A) cells. The root of *R. rosea* was extracted with ethanol and partitioned with water, ethyl acetate, and *n*-butanol fractions to evaluate acetylcholinesterase (AChE) inhibitory activity and neuroprotective activity. The ethyl acetate fraction exhibited the highest values of AChE inhibitory activity (49.2% ± 3%) and cell viability (50.7% ± 4.8%) for neuroprotection. The structure identification of the most potential fraction (ethyl acetate fraction) revealed 15 compounds, consisting of three tannins, five flavonoids, and seven phenolics by infrared spectroscopy, nuclear magnetic resonance, and mass spectroscopy. All compounds were evaluated for their neuroprotective activity. Salidroside had the most potential neuroprotective activity. Gallic acid and methyl gallate had potential cytotoxicity in N2A cells. This study showed that *R. rosea* might have potential neuroprotective activities.

## 1. Introduction

Alzheimer’s disease (AD) is the most common cause of dementia among older adults. AD is an irreversible, progressive brain disorder that slowly destroys memory and thinking skills. So far, all drug developments for AD have failed; thus, developing effective drugs is urgent.

Methylglyoxal (MG) is a reactive dicarbonyl intermediate and the most potent precursor for the formation of advanced glycation end-products (AGEs) [1]. AGEs are stable end-products formed in cells, and the accumulation of AGEs in the brain might contribute to neuronal inflammation [2]. Therefore, MG is an important factor in oxidative stress and neurodegeneration [3]. The brain has very high energy requirements and high glucose demand in order to maintain neural cell function. Such high levels of glucose uptake and glycolysis inevitably generate MG as by-products. Long-term exposure to MG induces neural progenitor cell death in vitro and impaired hippocampal neurogenesis, or it causes behavioral and biochemical alterations in rat brain [4,5]. Consequently, the MG adduct Nε-(carboxyethyl)lysine and its autoantibodies were increased in a certain neurodegenerative patient’s blood [6]. The glyoxalase system, an ubiquitous enzymatic pathway in eukaryotic cells, is responsible for detoxifying MG and, thus, plays a pivotal role in cellular defense against glycation and oxidative stress [7].

*Rhodiola rosea* L. (golden root, Arctic root, or Arctic rose) is a common member of the sedum and known as one of the most important and popular medicinal plants. *R. rosea* was discovered and studied for therapeutic purposes and accepted as an adaptogen [8]. *R. rosea* extract treatment for 24 h increased cell survival (human cortical cell line) significantly and prevented plasma membrane damage and morphological disruption caused by an oxidative stressor (i.e., H₂O₂) [9]. *R. rosea* root has a wide range of pharmacological activities, such as antioxidation, anti-inflammation [10], anti-cancer [11], cardioprotection [12], and neuroprotection [13], due to the existence of different secondary metabolites such as phenylethanoids, phenylpropanoids, flavonoids, flavolignans, phenolic acids, essential oil (monoterpenes), and cyanogenic glucosides, as well as polysaccharides and oligomeric proanthocyanidins [14,15,16]. It ameliorates oxidative stress, neuroinflammation, and excitotoxicity in brain tissues, and it has antagonistic effects of carcinogen-activated kinases [15]. However, no reports demonstrated the detailed neuroprotective activities of secondary metabolites to date. Therefore, this study aims to evaluate the neuroprotective activities of compounds extracted from the root of *R. rosea* against methylglyoxal (MG)-induced apoptosis in neuro-2A (N2A) cells.

## 2. Results

### 2.1. Acetylcholinesterase Inhibitory Activity

**Figure 1:**
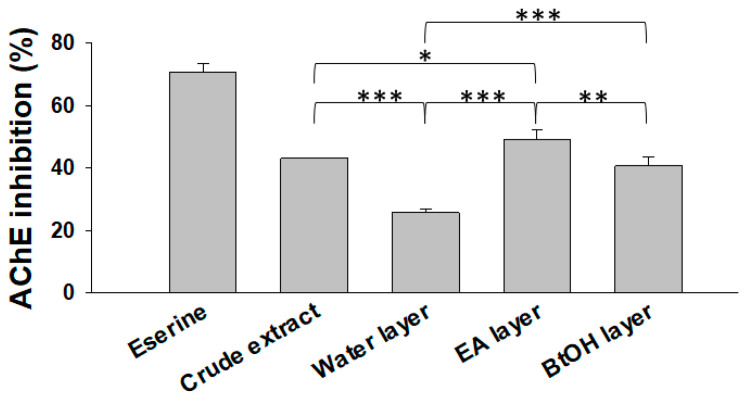
Percentage inhibition of acetylcholinesterase (AChE) activity of *Rhodiola rosea* root extract and fraction layers. The inhibitory activity was tested using the Ellman method by adding 500 μg/mL extract or a fraction layer, or 2.75 μg/mL eserine. Results are expressed as percentage control and data mean ± SD. Three sets of experiment were performed. EA, ethyl acetate; BtOH, *n*-butanol. * *p*-value < 0.05, ** *p*-value < 0.01, *** *p*-value < 0.001 compared with one another except eserine.

Maintaining acetylcholine levels due to inhibiting acetylcholinesterase (AChE) activity correlates with an improvement in neurodegeneration. AChE is an attractive target and considered a therapeutically relevant strategy for drug design and discovery for the treatment of peripheral diseases, such as Alzheimer’s disease [17,18]. Although *R. rosea* is shown to have a neuroprotective effect [19], the detailed activities remain unclear. Our research focused on activity-tracking strategies to analyze different parts of *R. rosea*. Our study showed that, at 500 μg/mL, the ethyl acetate fraction of *R. rosea* roots had the highest AChE inhibitory activity (49.2% ± 3%) compared to water and *n*-butanol fraction layers (Figure 1).

### 2.2. Neuroprotective Activity

**Figure 2:**
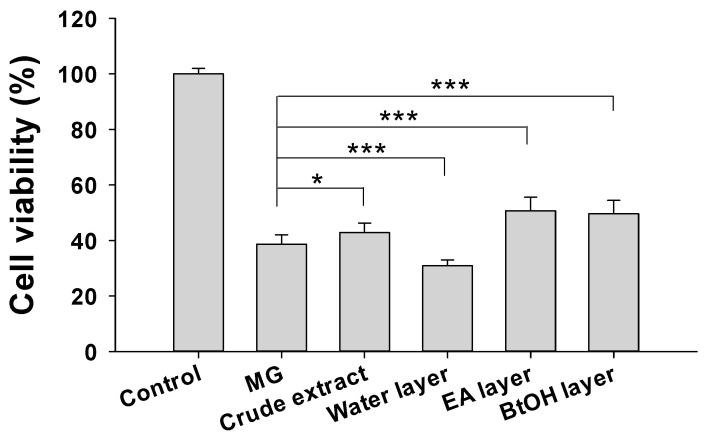
Neuroprotective activity of *R. rosea* extract and various layers. A concentration of 100 μg/mL *R. rosea* extract, water layer, or *n*-butanol (BtOH) layer, or 50 μg/mL ethyl acetate (EA) layer was used against 3.5 mM methylglyoxal (MG) in N2A cells. The cell viability was tested using the WST-8 method. Results are expressed as percentage control and data mean ± SD. Five sets of experiments were performed. * *p*-value < 0.05, *** *p*-value < 0.001 compared with the MG-treated group.

In addition to evaluating the effects of *R. rosea* root extract and fraction layers on AchE inhibition, we further investigated their neuroprotective effects on MG-induced cellular damage. Neuroprotection studies were performed on different cell lines, including SH-SY5Y human neuroblastoma cells [20], N2A mouse neuroblastoma cells [21,22], murine BV2 microglia cells [19,23], and PC-12 neuro-like cells [24]. The N2A cell line was applied extensively to screen compounds for neurotoxic/neuroprotective properties [21,25,26]. In this study, N2A cells were toxified by 3.5 mM MG to evaluate the neuroprotective activity of the different fraction layers from *R. rosea*. It was found that the cell viability was lowered to 38.7% ± 3.4% by MG. Either the ethyl acetate or the water layer at 100 μg/mL had cytotoxicity. In contrast, the ethyl acetate layer at 50 μg/mL showed the highest neuroprotective activity with 50.7% ± 4.8% cell viability followed by a 100 μg/mL *n*-butanol fraction layer (49.7% ± 4.8%) (Figure 2). Some compounds in the ethyl acetate layer are suspected to be toxic, while other compounds may have neuroprotective effects. Therefore, the neuroprotective activity of the ethyl acetate layer was further studied. The secondary metabolite composition was used in subsequent experiments.

### 2.3. Compounds Isolated from the Ethyl Acetate Layer of R. rosea Root

**Figure 3:**
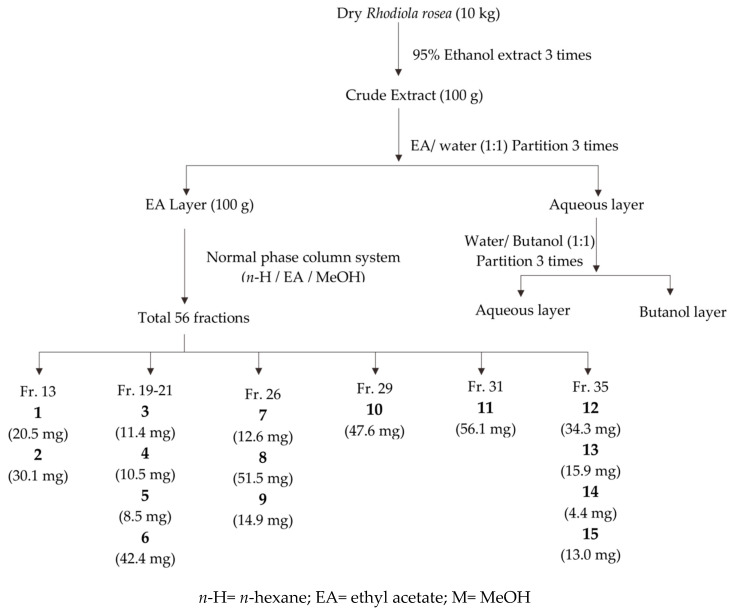
Bioactivity-guided fractionation and isolation of the ethanol extract of the root of *R. rosea* resulted in the isolation of 15 compounds.

**Figure 4:**
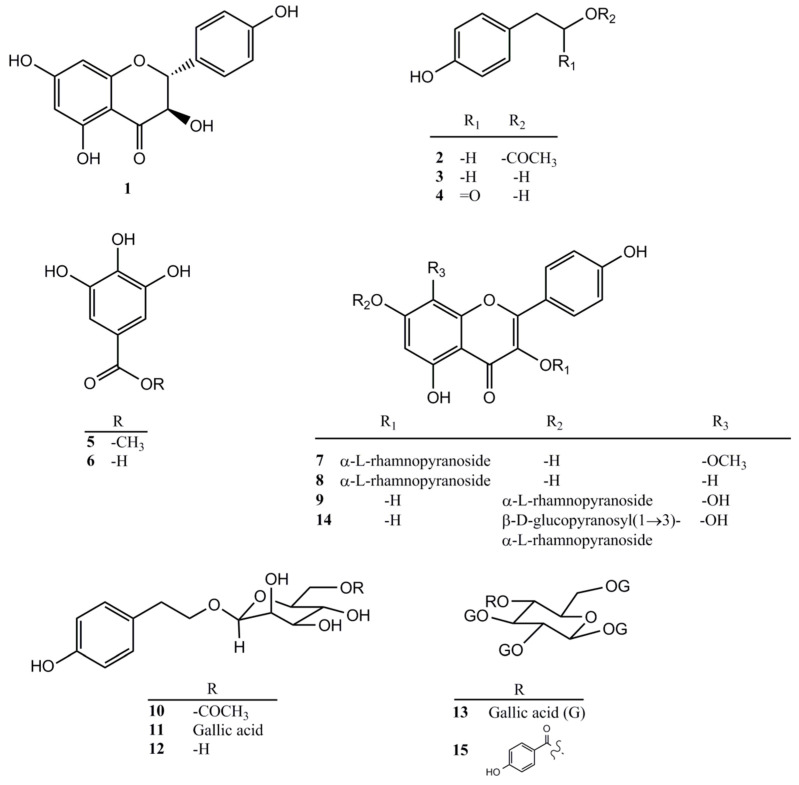
Structures of compounds **1–15**.

**Figure 5:**
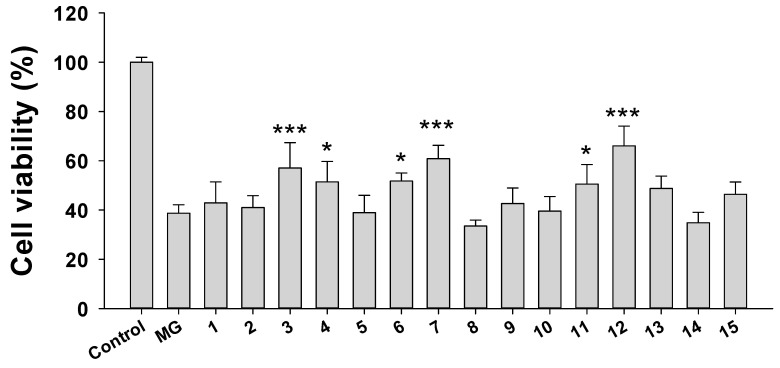
Neuroprotective activity of isolated compounds. The viability of N2A cells treated with 3.5 mM MG and 50 μM of each compound for 24 h was tested using the WST-8 method. Results are expressed as percentage control and data mean ± SD. Four sets of experiment were performed. * *p*-value < 0.05, *** *p*-value < 0.001 compared with the group treated with 3.5 mM MG.

To ascertain the cytoprotective constituents in *R. rosea* root, we performed a bio-guided purification strategy. The bio-guided fractionation and isolation flowchart (Figure 3) of the ethanol extract of the root of *R. rosea* resulted in the isolation of 15 compounds (Figure 4). Their structures were elucidated by co-chromatography with one-dimensional and two-dimensional (1D and 2D) NMR, mass spectrometric spectroscopic data analysis, and comparison to the literature. This fraction contained 15 compounds consisting of a flavonoid group, namely, aromadendrin (**1**) [27], a phenolic group (C6-C2), namely, 2-(4-hydroxyphenyl)ethyl acetate (**2**) [28], tyrosol (**3**) [28], 1-*O*-(4-hydroxyphenethyl)-β-D-glucopyranoside 6-acetate (**10**) [29], and salidroside (**12**) [30], a phenolic group (C6-C1), namely, 4-hydroxyphenylacetic acid (**4**), methyl gallate (**5**), and gallic acid (**6**) [31], a flavonoid glycoside group, namely, kaempferol-8-methoxy-3-*O*-α-L-rhamnopyranoside (**7**) [32], rhodiosin (**14**) [33], kaempferol-3*-O*-α-L-rhamnopyranoside (**8**) [34], and rhodionine (**9**) [33], and a tannin group, namely, 6’-*O*-galloyl salidroside (**11**) [35], 1,2,3,4,6-penta-*O*-galloyl-β-D-glucopyranoside (**13**) [36], and 1,2,3,6-tetra-*O*-galloyl-4-(4-hydroxy-benzoic acid)-β-D-glucopyranoside (**15**) [36]. All compounds were evaluated for neuroprotective activity by measuring the viability of N2A cells treated with 3.5 mM MG. Six compounds (**3**, **4**, **6**, **7**, **11**, and **12**) have potent neuroprotective activity, while compounds **5** and **8** have some cytotoxicity (Figure 5).

## 3. Discussion

*R. rosea* is reported to attenuate age-related declines and have anti-aging effects [37]. In this study, the root of *R. rosea* was extracted and partitioned to evaluate AChE inhibitory activity and neuroprotective activity. The evaluation of AChE inhibition activities, as well as anti-glycation and reactive carbonyl species scavenging capacities, is an element of neuroprotective potential selection strategies [38]. The ethyl acetate fraction exhibited the best activities. The structure identification of the ethyl acetate fraction revealed 15 compounds, consisting of tannins, flavonoids, and phenolics. Its ethanol extract had a protective effect on the neurotoxicity of N2A cells treated with MG, while the ethyl acetate layer had obvious effects on both AChE inhibitory activity and N2A cell protection. Previous studies demonstrated that *R. rosea* displayed AChE inhibitory activity and identified several active components [39,40]. Tyrosol was reported to protect N2A cells against amyloid β-induced toxicity [22]. Salidroside is one of the main ingredients of *R. rosea*, and it is reported to have good neuroprotective activity [41,42]. However, salidroside and other *R. rosea* components are also reported to exhibit some cytotoxicity against lung cancer cells, glioma cells, and sarcoma cells [43,44,45].

Compounds **3**, **4**, and **12** are phenolics, which are shown to be anti-inflammatory, antioxidant, and neuroprotective [46]. The structures of **3**, **4**, **11**, and **12** have similar skeletal tyrosol bones, suggesting that the tyrosol skeleton may play a role in neuroprotective activity [47,48]. Compounds **6** and **11** are compounds that have a partial structure of gallic acid, which has good neuroprotective activity. Compounds **13** and **15** are structured with multiple gallic acids, but their neuroprotective activities were reduced. It is speculated that excessive gallic acid might reduce neuroprotective activity [49]. Gallic acid (**6**), as well as methyl gallate (**5**), a methylated product of gallic acid, had potent cytotoxicity. Therefore, we speculate that compound **6** might be both neuroprotective and cytotoxic.

## 4. Materials and Methods

### 4.1. General Experimental Procedures

Semipreparative HPLC experiments were performed using a P230 HPLC Pump (Chrom Tech Inc., Apple Valley, MN, USA), RI detector (Bischoff Chromatography, Stuttgart, Germany), and columns of nucleodur C18 HTEC 250/10, 5 μm (Macherey-Nagel, Düren, Germany) and Syncronis C18 250/10 5 μm (Thermo Fisher Scientific Inc., Waltham, MA, USA). Thin-layer chromatography (TLC) was performed using Silica gel 60 RP-18 F254S (Merck, Darmstadt, Germany). NMR spectra were recorded using Bruker AV-500 MHz and Bruker AV-300 MHz spectrometers (Bruker, Rheinstetten, Germany). Infrared radiation (IR) spectra were recorded on a Jasco FT/IR 4100 Spectrometer (Jasco, Tokyo, Japan). Electrospray ionization mass spectrometry (ESIMS) data were acquired using an Orbitrap Elite mass spectrometer (Thermo Fisher Scientific Inc., USA). The polarization data were generated using a Jasco P-1020 Digital Polarimeter (Jasco, Tokyo, Japan). Enzymes and reagents used in the inhibition of AChE included *Electrophorus electricus* (electric eel)-derived AChE (Sigma, St. Louis, MO, USA), acetylthiocholine iodide (ATCI) (Sigma, USA), 5,5′-dithiobis (2-nitrobenzoic acid) (DTNB) (Sigma, USA), dimethyl sulfoxide (DMSO) (Sigma, USA), and eserine free base (Sigma, USA). The absorbance was measured using a microplate reader (Molecular Devices, San Jose, CA, USA). Cell viability and cytotoxicity were assayed using the WST-8 method with CCK-8 solution. All solvents were analytical grade.

### 4.2. Plant Materials

Dried roots of *R. rosea* L. were provided by Yong Chung Prosperous Biotech Co. Ltd., New Taipei city, Taiwan, in the summer of 2015. A voucher specimen (No. TMU060615) was identified by one of the authors (C.K.L.) and was deposited at the School of Pharmacy, Taipei Medical University, Taipei, Taiwan.

### 4.3. Extraction and Isolation of Compounds

Dried roots of *R. rosea* (10 kg) were extracted with 95% ethanol (three times) at room temperature. The solvent of filtrate was evaporated under reduced pressure to generate ethanol crude extract. The ethanol crude extract was then suspended in water and partitioned successively with ethyl acetate and *n*-butanol. The ethyl acetate fraction (100 g) was fractionated by normal-phase chromatography (NPC) over silica gel eluted with ethyl acetate, *n*-butanol, and methanol using a gradient system to collect 48 fractions (Fr.1–Fr.48). All compounds were obtained from Fr.13, 19, 20, 21, 26, 29, 31, and 35 by semipreparative HPLC. Compounds **1** (tR = 13.9 min) and **2** (tR = 20.2 min) were obtained from Fr.13 eluted with water/acetonitrile (2:1). Three compounds (**7** tR = 22.16 min, **8** tR = 24.36 min, and **9** tR = 29.48 min) were obtained from Fr. 26 eluted with water/acetonitrile (3:1). Two compounds (**10** tR = 9.18 min and **6** tR = 9.98 min) were from Fr.29 and 21 eluted with water/acetonitrile (3.25:1) and water/methanol (4:1), respectively. Fr.19 was separated by water/methanol (4:1) to collect three fractions (Fr. 19-1, 19-2, and 19-3), then Fr. 19-3 (tR = 15.30 min) was further separated by water/acetonitrile (5:1) to obtain compounds **3** (tR = 13.72 min) and **4** (tR = 16.46 min). Fractions Fr.20 and Fr.31 were separated by water/methanol (respectively, 4:1 and 5:2) to give Fr.20-2 (tR = 22.59 min) and Fr.31-2 (tR = 25.24 min), which were further separated by water/acetonitrile (respectively, 5:1 and 7:2) to give compound **5** and **11** (tR = 15.21 min) (tR = 11.22 min). From Fr.35, compound **12** (tR = 12.90 min) and residue were obtained with a water/methanol (5:2) elution, and then the residue was extracted with 100% methanol to give Fr.35 which was then separated with water/acetonitrile to collect compounds **13**, **14**, and **15** (tR = 10.34, 21.91, and 24.42 min, respectively).

### 4.4. Acetylcholinesterase Inhibitory Activity Assay

The AChE inhibitory activity assay was performed following the colorimetric method of Ellman [50] with the enzyme from *Electrophorus electricus*. In a 96-well microplate, 200 µL of test solution was applied in total. The test solution contained 100 µL of 1.5 mM 5,5′-dithiobis (2-nitrobenzoic acid) in 0.1 M sodium phosphate buffer (pH 8), 40 µL of 50 mM Tris-HCL buffer (pH = 8), 20 µL of 1.5 mM acetylthiocholine iodide, 20 µL of 500 µg/mL sample in Tris-HCL buffer with 1% DMSO, and 20 µL of 0.03 unit/mL AChE. The reaction was initiated by the addition of AChE, followed by colorimetric detection performed at 405 nm at a constant temperature of 37 °C for 10 min. The control without compound treatment was assigned as 100%, and the compound-treated groups were calculated relative to the control.

### 4.5. Cell Line and Culture

N2A cells (ATCC, CCL-131) were maintained in minimum essential medium (Eagle) with 2 mM L-glutamine, 0.1 mM non-essential amino acids, 1.5 g/L sodium bicarbonate, and 1.0 mM sodium pyruvate (Life Technologies, Carlsbad, CA, United States), supplemented with 10% heat-inactivated fetal bovine serum (Life Technologies), 100 units/mL penicillin, 100 μg/mL streptomycin, and 2.5 μg/mL amphotericin B (Life Technologies) as indicated [51,52]. Cultures were incubated at 37 °C with 5% CO_2_ in a humidified incubator.

### 4.6. Cell Treatment

The N2A cells were seeded at a density of 10,000 cells/well in a 96-well plate in a cell culture chamber for 24 h. Then, the culture medium was replaced with that containing 3.5 mM MG and various concentrations of *R. rosea* extract, water layer, *n*-butanol layer, ethyl acetate layer, or 50 μM isolated compound. Cells were cultured for another 24 h and then analyzed for cell viability.

### 4.7. Assessment of Cell Viability

Cell viability was determined using cell counting kit-8 (CCK-8) (Sigma, USA). Ten microlitres of CCK-8 solution and 100 μL of the culture solution were added to each well of a 96-well plate. Cells were incubated at 37 °C, 5% CO_2_, and 95% relative humidity for 2 h. After the end of the incubation time, the absorbance of each well was measured at 460 nm. The cell viability was calculated by normalizing with the absorbance of the control group without compounds.

### 4.8. Statistical Analyses

Experiments were performed in triplicate, and the results were expressed as mean ± SD. Statistical evaluation of the data was performed with one-way ANOVA using SigmaPlot 14 (Systat Software Inc. San Jose, CA, USA). A value of *p* < 0.05 was considered statistically significant.

## 5. Conclusions

*R. rosea* has the effect of “extending life”. The market effect of *Rhodiola rosea* health food is related to anti-aging. Its ethanol extract has a protective effect against the neurotoxicity induced by methylglyoxal on Neuro-2A cells. Through a bioactivity guide fractionation and isolation strategy, we found that the ethyl acetate layer exerted the highest AChE inhibitory activity (49.2% ± 3%) and neuroprotective activity (50.7% ± 4.8% cell viability). Further purification of ethyl acetate layer revealed 15 components. Salidroside is one of the main ingredients of *R. rosea*, and it is reported to have good neurocyte protective activity. We obtained the same results in this study. In addition, compounds featured a tyrosol backbone including tyrosol (**3**), 4-hydroxyphenylacetic acid (**4**), 6’-*O*-galloyl salidroside (**11**), and salidroside (**12**), or compounds possessing a single gallic acid moiety including gallic acid (**6**) and 6’-*O*-galloyl salidroside (**11**), which may exhibit important neurocyte protective activities. Compounds containing multiple gallic acid moieties including 1,2,3,4,6-penta-*O*-galloyl-β-D-glucopyranoside (**13**) and 1,2,3,6-tetra-*O*-galloyl-4-(4-hydroxy-benzoic acid)-β-D-glucopyranoside (**15**) are speculated to have greater cytotoxicity and poor neuroprotective activity. The active components merit further exploration in the development of potential drugs for neurodegenerative disease.

## References

[1] Schalkwijk C.G., Stehouwer C.D.A. (2019). Methylglyoxal, a highly reactive dicarbonyl compound, in diabetes, Its vascular complications, and other age-related diseases. Physiol. Rev..

[2] Wetzels S., Vanmierlo T., Scheijen J.L.J.M., Horssen J.V., Amor S., Somers V., Schalkwijk C.G., Hendriks J.J.A., Wouters K. (2019). Methylglyoxal-derived advanced glycation endproducts accumulate in multiple sclerosis lesions. Front. Immunol..

[3] Frandsen J.R., Narayanasamy P. (2018). Neuroprotection through flavonoid: Enhancement of the glyoxalase pathway. Redox Biology.

[4] Hansen F., Pandolfo P., Galland F., Torres F.V., Dutra M.F., Batassini C., Guerra M.C., Leite M.C., Gonçalves C.A. (2016). Methylglyoxal can mediate behavioral and neurochemical alterations in rat brain. Physiol. Behav..

[5] Chun H.J., Lee Y., Kim A.H., Lee J. (2016). Methylglyoxal causes cell death in neural progenitor cells and impairs adult hippocampal neurogenesis. Neurotox. Res..

[6] Lin C.Y., Sheu J.J., Tsai I.S., Wang S.T., Yang L.Y., Chang H.W., Lee H.M., Kao S.H., Lee C.K., Chenn C.H. (2018). Elevated IgM against Nε-(Carboxyethyl)lysine-modified Apolipoprotein A1 peptide 141–147 in Taiwanese with Alzheimer’s disease. Clin. Biochem..

[7] Allaman I., Bélanger M., Magistretti P.J. (2015). Methylglyoxal, the dark side of glycolysis. Front. Neurosci..

[8] Khanna K., Mishra K.P., Ganju L., Singh S.B. (2017). Golden root: A wholesome treat of immunity. Biomed. Pharmacother..

[9] Palumbo D.R., Occhiuto F., Spadaro F., Circosta C. (2012). *Rhodiola rosea* extract protects human cortical neurons against glutamate and hydrogen peroxide-induced cell death through reduction in the accumulation of intracellular calcium. Phytother. Res..

[10] Pu W.I., Zhang M.Y., Bai R.Y., Sun L.K., Li W.H., Yu Y.L., Zhang Y., Song L., Wang J.X., Peng Y.F. (2020). Anti-inflammatory effects of *Rhodiola rosea* L.: A review. Biomed. Pharmacother..

[11] Liu Z., Li X., Simoneau A.R., Jafari M., Zi X. (2012). *Rhodiola rosea* extracts and salidroside decrease the growth of bladder cancer cell lines via inhibition of the mTOR pathway and induction of autophagy. Mol. Carcinogen..

[12] Zhang J., Liu A., Hou R., Zhang J., Jia X., Jiang W., Chen J. (2009). Salidroside protects cardiomyocyte against hypoxia-induced death: A HIF-1α-activated and VEGF-mediated pathway. Eur. J. Pharm..

[13] Jacob R., Nalini G., Chidambaranathan N. (2013). Neuroprotective effect of *Rhodiola rosea* Linn against MPTP induced cognitive impairment and oxidative stress. Ann. Neurosci..

[14] Panossian A., Wikman G., Sarris J. (2010). Rosenroot (*Rhodiola rosea*): Traditional use, chemical composition, pharmacology and clinical efficacy. Phytomedicine.

[15] Nabavi S.F., Braidy N., Orhan I.E., Badiee A., Daglia M., Nabavi S.M., Rhodiola rosea L., *Rhodiola rosea* L (2016). and Alzheimer’s Disease: From Farm to Pharmacy. Phytother. Res..

[16] Zhou T., Zheng J., Zhou Y., Li S., Gan R.Y., Li H.B., Gan R.Y. (2015). Chemical components and bioactivities of *Rhodiola rosea*. Int. J. Tradit. Nat. Med..

[17] Lima B.R., Lima J.M., Maciel J.B., Valentim C.Q., Nunomura R.C.S., Lima E.S., Koolen H.H.F., Souza A.D.L., Pinheiro M.B., Cass Q.B. (2019). Synthesis and inhibition evaluation of new benzyltetrahydroprotoberberine alkaloids designed as acetylcholinesterase inhibitors. Front. Chem..

[18] Santos T.C., Gomes T.M., Pinto B.A.S., Camara A.L., Paes A.M.A. (2018). Naturally occurring acetylcholinesterase inhibitors and their potential use for Alzheimer’s disease therapy. Front. Pharmacol..

[19] Lee Y., Jung J.C., Jang S., Kim J., Ali Z., khan I.A., Oh S. (2013). Anti-Inflammatory and neuroprotective effects of constituents isolated from *Rhodiola rosea*. Evid-Based Complement. Altern. Med..

[20] Sharma M.K., Jalewa J., Hölscher C. (2014). Neuroprotective and anti-apoptotic effects of liraglutide on SH-SY5Y cells exposed to methylglyoxal stress. J. Neurochem..

[21] Huang S.M., Chuang H.C., Wu C.H., Yen G.C. (2008). Cytoprotective effects of phenolic acids on methylglyoxal-induced apoptosis in Neuro-2A cells. Mol. Nutr. Food Res..

[22] Thibault C.S.L., Arseneault M., Longpre F., Ramassamy C. (2011). Tyrosol and hydroxytyrosol two main components of olive oil, protect N2a cells against Amyloid-β-Induced toxicity. Involvement of the NF-κB signaling. Curr. Alzheimer Res..

[23] Wang Y.M., Ming W.Z., Liang H., Wang Y.J., Zhang Y.H., Meng D.L. (2020). Isoquinolines from national herb *Corydalis tomentella* and neuroprotective effect against lipopolysaccharide-induced BV2 microglia cells. Bioorganic Chem..

[24] Lai M.C., Liu W.Y., Liou S.S., Liu I.M. (2020). The protective effects of moscatilin against methylglyoxal-induced neurotoxicity via the regulation of p38/JNK MAPK pathways in PC12 neuron-like cells. Food Chem. Toxicol..

[25] Cásedas G., Les F., Choya-Foces C., Hugo M., López V. (2020). The metabolite Urolithin-A ameliorates oxidative stress in Neuro-2a cells, becoming a potential neuroprotective agent. Antioxidants.

[26] Chakraborty J., Rajamma U., Jana N., Mohanakumar K.P. (2015). Quercetin improves the activity of the ubiquitin-proteasomal system in 150Q mutated huntingtin-expressing cells but exerts detrimental effects on neuronal survivability. J. Neurosci. Res..

[27] Liao C.R., Kuo Y.H., Ho Y.L., Wang C.Y., Yang C.S., Lin C.W., Chang Y.S. (2014). Studies on cytotoxic constituents from the leaves of *Elaeagnus oldhamii* maxim. in non-small cell lung cancer A549 cells. Molecules.

[28] Mateos R., Espartero J.L., Trujillo M., Rıos J.J., Camacho M.L., Alcudia F., Cert A. (2001). Determination of phenols, flavones, and lignans in virgin olive oils by solid-phase extraction and high-performance liquid chromatography with diode array ultraviolet detection. J. Agric. Food Chem..

[29] Yu H.L., Xu J.H., Wang Y.X., Lu W.Y., Lin G.Q. (2008). Assembly of a three-dimensional array of glycoconjugates by combinatorial biocatalysis in nonaqueous media. J. Comb. Chem..

[30] Guney T., Kohles S.A., Thompson V.L., Phillips G.J., Kraus G.A. (2015). Heterocycles from wine: Synthesis and biological evaluation of salidrosides. Tetrahedron.

[31] Sudjaroen Y., Hull W.E., Erben G., Würtele G., Changbumrung S., Ulrich C.M., Owen R.W. (2012). Isolation and characterization of ellagitannins as the major polyphenolic components of longan (*Dimocarpus longan* Lour) seeds. Phytochemistry.

[32] An J.P., Dang L.H., Ha T.K.Q., Pham H.T.T., Lee B.W., Lee C.H., Oh W.K. (2019). Flavone glycosides from *Sicyos angulatus* and their inhibitory effects on hepatic lipid accumulation. Phytochemistry.

[33] Jeong H.J., Ryu Y.B., Park S., Kim J.H., Kwon H.J., Kim J.H., Park K.H., Rho M.C., Lee W.S. (2009). Neuraminidase inhibitory activities of flavonols isolated from *Rhodiola rosea* roots and their in vitro anti-influenza viral activities. Bioorg. Med. Chem..

[34] Ahmed A.A., Mabry T.J., Matlin S.A. (1989). Flavonoids of the flowers of *Silybum marianum*. Phytochemistry.

[35] Nonaka G., Nishimura H., Nishioka I. (2011). Tannins and related compounds. IV. Seven new phenol glucoside gallates from *Quercus stenophylla* Makino. Chem. Pharm. Bull..

[36] Mageed W.M.A., Bayoumi S.A.H., Chen C., Vavricka C.J., Li L., Malik A., Dai H., Song F., Wang L., Zhang J. (2014). Benzophenone C-glucosides and gallotannins from mango tree stem bark with broad-spectrum anti-viral activity. Bioorg. Med. Chem..

[37] Zhuang W., Yue L., Dang X., Chen F., Gong Y., Lin X., Luo Y. (2019). Rosenroot (Rhodiola): Potential applications in Aging-related diseases. Aging Dis..

[38] Liu W., Ma H., Dasilva N.A., Rose K.N., Johnson S.L., Zhang L., Wan C., Dain J.A., Seeram N.P. (2016). Development of a neuroprotective potential algorithm for medicinal plants. Neurochem. Int..

[39] Hillhouse B., Ming D.S., French C., Towers G.H. (2004). Acetylcholine esterase inhibitors in *Rhodiola rosea*. Pharm. Biol..

[40] Wang H., Zhou G., Gao X., Wang Y., Yao W. (2007). Acetylcholinesterase inhibitory-active components of *Rhodiola rosea* L.. Food Chem..

[41] Zhang L., Yu H., Sun Y., Lin X., Chen B., Tan T., Cao G., Wang Z. (2007). Protective effects of salidroside on hydrogen peroxide-induced apoptosis in SH-SY5Y human neuroblastoma cells. Eur. J. Pharmacol..

[42] Tao K., Wang B., Feng D., Zhang W., Lu F., Lai J., Huang L., Nie T., Yang Q. (2016). Salidroside protects against 6-hydroxydopamine-induced cytotoxicity by attenuating ER stress. Neurosci. Bull..

[43] Cai J., Li W., Wang H., Yan W., Zhou Y., Wang G., Cui J., Wang F. (2012). Antitumor effects of a purified polysaccharide from *Rhodiola rosea* and its action mechanism. Carbohydr. Polym..

[44] Wang J., Li J.Z., Lu A.X., Zhang K.F., Li B.J. (2014). Anticancer effect of salidroside on A549 lung cancer cells through inhibition of oxidative stress and phospho-p38 expression. Oncol. Lett..

[45] Zhang Y., Yao Y., Wang H., Guo Y., Zhang H., Chen L. (2013). Effects of salidroside on glioma formation and growth inhibition together with improvement of tumor microenvironment. Chin. J. Cancer Res..

[46] Spagnuolo C., Napolitano M., Tedesco I., Moccia S., Milito A., Russo G.L. (2016). Neuroprotective role of natural polyphenols. Curr. Top. Med. Chem..

[47] Marković A.K., Torić J., Barbarić M., Brala C.J. (2019). Hydroxytyrosol, tyrosol and derivatives and their potential effects on human health. Molecules.

[48] Atochin D.N., Chernysheva G.A., Smolyakova V.I., Osipenko A.N., Logvinov S.V., Zhdankina A.A., Sysolyatin S.V., Kryukov Y.A., Anfinogenova Y., Plotnikova T.M. (2016). Neuroprotective effects of *p*-tyrosol after the global cerebral ischemia in rats. Phytomedicine.

[49] Park W., Chang M.S., Kim H., Choi H.Y., Yang W.M., Kim D.R., Park E.H., Park S.K. (2008). Cytotoxic effect of gallic acid on testicular cell lines with increasing H_2_O_2_ level in GC-1 spg cells. Toxicol. In Vitro.

[50] Ellman G.L., Courtney K.D., Andres V., Featherstone R.M. (1961). A new and rapid colorimetric determination of acetylcholinesterase activity. Biochem. Pharmacol..

[51] Tsai Y.F., Yang D.J., Ngo T.H., Shih C.H., Wu Y.F., Lee C.K., Phraekanjanavichid V., Yen S.F., Kao S.H., Lee H.M. (2019). Ganglioside Hp-s1 analogue inhibits amyloidogenic toxicity in Alzheimer’s disease model cells. ACS Chem. Neurosci..

[52] Ting L.L., Lu H.T., Yen S.F., Ngo T.H., Tu F.Y., Tsai I.S., Tsai Y.H., Chang F.Y., Li X.Z., Li S. (2019). Expression of AHI1 rescues amyloidogenic pathology in Alzheimer’s disease model cells. Mol. Neurobiol..

